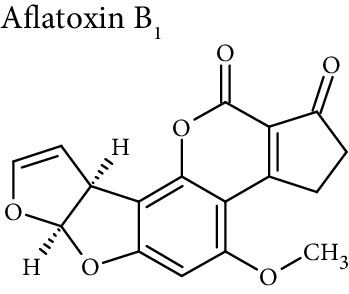# Analytical Strategies in Lipidomics for Discovery of Functional Biomarkers from Human Saliva

**DOI:** 10.1155/2019/6741518

**Published:** 2019-12-04

**Authors:** Snezana Agatonovic-Kustrin, David William Morton, Valeriy Smirnov, Alexey Petukhov, Vladimir Gegechkori, Vera Kuzina, Natalya Gorpinchenko, Galina Ramenskaya

**Affiliations:** ^1^Department of Pharmaceutical and Toxicological Chemistry Named after Arzamastsev of the Institute of Pharmacy, I.M. Sechenov First Moscow State Medical University (Sechenov University), Trubetskaya, Moscow 119991, Russia; ^2^Department of Pharmacy and Biomedical Sciences, La Trobe Institute for Molecular Sciences, La Trobe University, Bendigo, Australia

## Abstract

Human saliva is increasingly being used and validated as a biofluid for diagnosing, monitoring systemic disease status, and predicting disease progression. The discovery of biomarkers in saliva biofluid offers unique opportunities to bypass the invasive procedure of blood sampling by using oral fluids to evaluate the health condition of a patient. Saliva biofluid is clinically relevant since its components can be found in plasma. As salivary lipids are among the most essential cellular components of human saliva, there is great potential for their use as biomarkers. Lipid composition in cells and tissues change in response to physiological changes and normal tissues have a different lipid composition than tissues affected by diseases. Lipid imbalance is closely associated with a number of human lifestyle-related diseases, such as atherosclerosis, diabetes, metabolic syndromes, systemic cancers, neurodegenerative diseases, and infectious diseases. Thus, identification of lipidomic biomarkers or key lipids in different diseases can be used to diagnose diseases and disease state and evaluate response to treatments. However, further research is needed to determine if saliva can be used as a surrogate to serum lipid profiles, given that highly sensitive methods with low limits of detection are needed to discover salivary biomarkers in order to develop reliable diagnostic and disease monitoring salivary tests. Lipidomic methods have greatly advanced in recent years with a constant advance in mass spectrometry (MS) and development of MS detectors with high accuracy and high resolution that are able to determine the elemental composition of many lipids.

## 1. Introduction

There is a constant search for more accurate and cost-effective diagnostic assessment methods. The components of the human body are closely interdependent, and disease conditions in some organs, or their components, can influence the development of disease in other parts of the body. Biofluids can provide information on the molecular changes that occur in the body in disease conditions. These disease-related changes in the molecules can then be used as biomarkers for screening and early-stage detection of the disease. The use of metabolomics as a diagnostic tool in samples of noninvasively collected biofluids such as breath, saliva, and urine that contain biomarker compounds shows great promise for diagnosis of disease [1]. Saliva is considered as an ultrafiltrate of plasma with good correlations between the concentration of many compounds in saliva and their concentration in the blood observed [2]. Therefore, there is a focus on developing analytical methods that use saliva as a diagnostic fluid.

Salivaomics is a new term used to describe saliva-based techniques that are used to investigate different types of compounds found in saliva [3]. The advantage is that saliva samples can be easily collected. Thus, it can be used in circumstances in which blood samples are more difficult to collect such as in children, anxious and handicapped patients, or in patients where blood drawing may be difficult (individuals with compromised venous access and patients with haemophilia).

Saliva is the most available biofluid with many functions in the oral cavity. It provides protection for the oral tissues against biological, mechanical, and chemical stimuli, allows the perception of taste and temperature, and is responsible for initial food digestion [4]. It is also involved in the maintenance of optimal conditions in the oral cavity, minimising bacterial growth, regulating pH, and neutralising acidic reflux in the oesophagus [5]. An adequate supply of saliva is essential for the maintenance of oral tissue providing lubrication and protective functions for tissues and mucous membrane surfaces, thereby allowing articulation and swallowing. These functions and properties of saliva are attributed to the presence of electrolytes, buffering agents, proteins, glycoproteins, and lipids. However, saliva's flow and chemical composition are affected by gender, age, daily circadian cycle, emotional state, hydration, physical exercise, medication, systemic diseases, substance abuse, and nutrition [6, 7]. The physiological importance of saliva has only been recently recognized with the finding that it contains many components that can be used as potential biomarkers [8]. Saliva biomarkers are analytes that can be used to detect oral and systemic disease states or to confirm the exposure to various harmful substances.

## 2. Saliva as a Diagnostic Biofluid

Saliva as a diagnostic biofluid offers advantages over serum for developing clinically validated diagnostic health tests. Saliva sampling is easy, noninvasive, and painless and therefore more acceptable to patients with fewer compliance problems compared to blood sampling. A large number of diagnostic analytes that are found in saliva are correlated with their levels in the blood suggesting that saliva is a promising biofluid for drug monitoring and for biomarker screening of [9, 10]. Analysis of saliva can be used for early detection, diagnosing, and monitoring the progression of oral diseases and systemic conditions, the effectiveness of treatment regimens [11], or exposure to harmful substances [12, 13]. This makes saliva an eligible biological fluid for studying diseases where the salivary glands are the primary target organs, such as the autoimmune disease of Sjögren's syndrome [14, 15].

Salivary excretion of some drugs can be used in pharmacokinetic studies to assess drug bioavailability [16], for therapeutic drug monitoring, and as an indicator of drug abuse. Most of the drugs in the blood are reversibly bound to plasma proteins and only the free fraction of the drug is pharmacologically active and can cross biological membranes to reach their target. Free molecules can also travel through the cells and into saliva ducts which can then be assayed in saliva samples. Thus, levels of drugs in saliva should reflect the concentration of the biologically active free fraction of the drug and provide clinically relevant information [17]. For example, saliva is the preferred sample biofluid for studies assessing nicotine exposure since concentrations of nicotine and its metabolites in saliva are correlated with their plasma concentrations [18, 19]. Un-ionized drugs, such as steroids, easily cross from plasma to saliva and offer a noninvasive monitoring method. Small molecules, like hormones, can be determined in saliva and used as indicators of health and disease status in humans [20, 21].

### 2.1. *Sialochemistry* (Chemical Analysis of Saliva) and Salivaomics (Salivary Diagnostics)

Human saliva is a highly complex mixture of compounds that originates from several sources; secretions of the major and minor salivary glands, sulcular or gingival crevicular fluid, oral bacteria and their metabolites, and food debris. Whole saliva contains a variety of biologically important compounds like enzymes, hormones, nucleic acids, peptides, proteins, metabolites, immunoglobulins, mucins, electrolytes, and nitrogen-containing compounds such as urea and ammonia [22]. Despite its complexity, it is a dilute fluid containing 99% water.

### 2.2. Saliva Biomarkers

Salivary proteins are well characterized. About 25% of salivary proteins are plasma components, while the rest comes from salivary glands and desquamated epithelial cells [23]. Thanks to proteomic studies, more than 2200 proteins in saliva have been identified, with some of them being identified as possible clinical biomarkers in occupational and environmental medicine [24]. Sayer et al. [25] have shown that the catalytic activity of acetylcholinesterase (AChE) in saliva is stable at room temperature for up to 6 hours. Measurement of AChE inhibition was used as a biomarker for organophosphate and carbamate pesticide poisoning. Henn [26] suggested that saliva AChE inhibition can be used to indicate potential neurotoxic effects following exposure to carbamate and organophosphorus pesticides.

Recently, a novel noninvasive method for stress assessment by monitoring salivary *α*-amylase has been developed [27, 28]. Salivary *α*-amylase is an important digestive enzyme found in the oral cavity that breaks down polysaccharides, starch, and glycogen into shorter oligomers (glucose and maltose), starting the digestive process. Its activity is induced by stress but independent of the salivary flow rate [28]. Although in vitro studies suggest that *α*-amylase release can also be induced by parasympathetic activation, *α*-amylase activity is not a reliable marker of catecholamine levels [28–30]. Hence, *α*-amylase activity is a general autonomic biomarker that can complement but not replace catecholamine levels and cardiac activity as a stress indicator.

Levels of cortisol in saliva, as a stress biomarker, offer a new research approach due to its easy collection and wide potential application [31, 32]. Cortisol measured from serum or plasma represents total cortisol, not the free, unbound, biologically active cortisol. There is also additional stress while taking blood samples that may lead to false-positive results. The concentrations of cortisol in saliva and unbound serum concentration correlate well, regardless of the serum protein concentrations [33]. Since the concentration of cortisol in the saliva is in equilibrium with the concentration of free cortisol in blood plasma, measurement of salivary cortisol in the evening (trough) and morning (peak) can be used as a simple screening test for Cushing's syndrome [34].

The effects of aerobic exercise in exercise and sport have also been investigated through monitoring steroid, peptide, and immune markers in saliva [35]. Levels of cortisol and testosterone in saliva were used as indicators of physical fitness and to detect the development of overtraining syndrome. Overtraining syndrome is a condition of decreased performance and fatigue in athletes, caused by metabolic, immune, hormonal, and other dysfunctions due to an imbalance between training stress and insufficient recovery time.

### 2.3. Salivaomics

**Figure 1:**
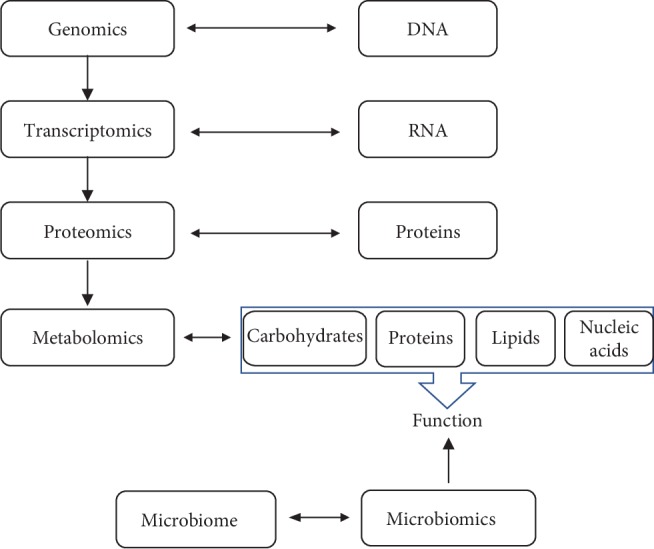
The relationship between genomics, transcriptomics, proteomics, metabolomics, and microbiomics.

The term “salivaomics” was introduced in 2008 to describe the metabolomic, proteomic, transcriptomic, and genomic constituents of saliva [36]. The word “omics” derives from the Greek suffix, “ome,” which means all, whole, or complete, refers to methods that study the global actions, roles, and relationships of the complete set of molecules in the biological system or organ. The area of bioscience that falls under the umbrella of “omics,” e.g. genomics, proteomics, transcriptomics, lipidomics, and metabolomics, aims to provide detailed profile characterizations which are characteristic of different human diseases (Figure 1). There is also microbiomics, research on the human microbiome. Humans are born with certain bacteria and viruses which develop further in response to different stimuli and environmental factors. Understanding the human microbiome and its role in health and disease and how they interact with their host is essential from a clinical perspective.

Saliva has been shown to be a valuable analytical tool in metabolomics [2]. More recently, lipidomics, the study of the global lipid profile (i.e., lipidome), has emerged as a new subfield of metabolomics. It provides a complete *analysis* (characterization and quantification), with information on the structure and function of lipids, and their interaction with other lipids, proteins, and metabolic pathways [37].

The lipidome of an organ can provide a fingerprint of the metabolic changes that occur during a disease and biomarkers for early detection of a disease. Comprehensive identification and precise quantification of lipids are expected to provide a powerful tool to diagnose and understand the mechanism of lipid-related diseases and disorders.

Lipids, proteins, carbohydrates, and nucleic acids are the four major classes of biomolecules that make up most of the materials in our cells. The primary role of lipids in the body is to form a membrane, as a permeability barrier, and to serve as energy source. However, lipids have many other essential biological roles. For example, the human brain is nearly 60% fat (by dry weight), being extremely rich in lipid and fatty acid-binding proteins. Apart from adipose tissue, the brain has the highest concentration of lipids of any organ. Such fats are linked to neuronal functions, including the insulating qualities of myelin, and provide the correct environment for protein function within a cell membrane.

Cellular lipids are generally highly complex structures with remarkable structural diversities in terms of length of the aliphatic chain, degree of unsaturation, position of double bonds, branching, etc. making thousands of different lipid species. LIPID MAPS, the most comprehensive lipid database, contains about 10,000 different lipid molecules [38]. Under the LIPID MAPS system, a unique 12- or 14-character identifier is introduced to represent each lipid molecule and to assist with lipid identification [39]. Despite their structural diversity, lipids are divided into eight primary categories (Table 1) according to the International Lipid Classification and Nomenclature Committee [40]. Each main lipid category is further divided into classes and subclasses leading to enormous number of lipid species [41]. Many of these classes contain subclasses with many unique and distinct molecules. For instance, prenol lipids are subdivided into classes (isoprenoids), subclasses (C15 isoprenoids or sesquiterpenes), and fourth-level subclasses (isabolane sesquiterpenoids). This structural diversity leads to large variations in their physical and chemical properties and various tissue/cellular molecular functions.

Saliva contains 5 major lipid groups; fatty acyls, glycerolipids, glycerophospholipids, sphingolipids, and sterol lipids. Recent work on the analysis of 305 saliva samples from 61 participants identified 424.1 ± 28.4 metabolites (497 metabolites in total) with the mass range of 75-862 Da. The mostly represented metabolites were amino acids and their derivatives (29.6% of total) and lipids (28.4%). Among the 141 lipid metabolites, the most common subclasses were phospholipids (34) and sphingolipids (24) [42].

### 2.4. Saliva Metabolomics

Salivary diagnostics can be used to develop diagnostic tests for early detection of oral diseases and their association with systemic conditions. Wang and coworkers identified 14 potential biomarkers for diagnosis of oral squamous cell carcinoma (OSCC) [43]. Combination of five salivary biomarkers (N-acetyl-L-phenylalanine, phytosphingosine, propionylcholine, sphinganine, and S-carboxymethyl-L-cysteine) produced reasonable accuracy (AUC = 0.997), sensitivity (100%), and specificity (96.7%) in differentiating early stages of OSCC from the control. Saddoughi and coworkers [44] reported that the increase in serum C18-ceramide level can be used as a new biomarker for response to chemotherapy with gemcitabine/doxorubicin to metastatic head and neck squamous cell carcinoma (HNSCC) patients.

Most theories propose that mediation of the inflammatory response is related to periodontal disease and other diseases that may be present in the body. For example, recent studies suggest that increased systemic inflammatory mediators in periodontal (gum) disease are related to systemic disorders [45] such as arteriosclerotic cardiovascular disease or metabolic syndrome [46]. Cardiovascular diseases are the leading cause of lethality, due to atherosclerosis, in developing and developed countries [47]. The increased incidence of periodontal (gum) disease in patients with atherosclerosis has been associated with the incidence of acute and chronic coronary heart disease [48]. Increased body weight, dyslipidaemia, and hypertension as individual components or combined in the metabolic syndrome are also associated with increased prevalence of periodontitis [47].

## 3. Lipidomics and Salivary Lipids

### 3.1. Lipid Composition in Response to Disease

Lipidomics research involves analysis and characterization of lipids and their function in different cell types, tissues, and biofluids. Lipids are essential cellular components used by cells to store and obtain energy, for structural purposes to form cell membranes, and as permeability barrier for cells and subcellular organelles. Lipids are also involved in several metabolic pathways [49], as signalling molecules, precursors for secondary messengers, in the regulation of cell growth, proliferation, differentiation, survival, apoptosis, inflammation, and other cell functions [50]. Lipid composition varies between cell and tissue types and may change in response to physiological changes. Because lipids and associated species are crucial cell and tissue components, many recent studies have demonstrated their important role in health and disease. Thus, normal tissues have a different lipid composition than tissues affected by diseases due to the changes in genetic status (variation in genes), epigenetic changes in gene expression, protein expression, and posttranslational modifications.

Identification of lipidomic biomarkers or key lipids in different diseases can be used to diagnose diseases and disease state and evaluate responses to treatments. Lipid imbalance is also closely associated with a number of human lifestyle-related diseases, such as atherosclerosis, diabetes, metabolic syndromes, systemic cancers [51], neurodegenerative diseases like Alzheimer's disease [52], and infectious diseases [53]. Salivary lipids are mostly secreted by the major salivary glands, but some lipids like cholesterol and some fatty acids diffuse directly from serum into saliva [48]. Qualitative and quantitative composition of lipids changes in various pathological states and human diseases. However, additional studies are required to determine the specificity of saliva as a surrogate for serum lipid profiles.

### 3.2. Salivary Lipids and Dental Caries

Lipid analysis of parotid saliva between two groups of female patients, one group prone to and one group resistant to dental caries, showed higher total lipid concentration in the group susceptible to caries [54]. Although the lipid composition remained the same, higher concentrations of neutral lipids, free fatty acids, and triglycerides that were observed in the group susceptible to caries are probably associated with the development of caries. An increase in total salivary protein was also observed in this group.

### 3.3. Saliva Biomarkers

Apolipoprotein E (Apo*ε*) is a glycoprotein involved in lipid transport with a significant role in lipid metabolism and regulating inflammation [55]. The gene for Apo*ε* is polymorphic, presenting with three common alleles: *ε*2, *ε*3, and *ε*4, at a single gene locus on chromosome 19. The presence of the Apo*ε*4 allele is associated with an increased risk of coronary heart disease and Alzheimer's disease [56]. A liquid chromatography-multiple reaction monitoring (LC-MRM) approach enabled detection and quantification of these 3 isoforms of Apo*ε* in human saliva. The sample preparation has been optimized and automatized to facilitate the phenotype interpretation and to allow for high throughput screening.

The use of malondialdehyde (MDA) as a biomarker for lipid peroxidation was investigated in chronic oral inflammatory conditions such as periodontitis. Increased levels of MDA were observed in the saliva of patients that had periodontitis and that were smokers compared to the nonsmoking control group [57]. Shetty and coworkers have compared the levels of MDA, as a lipid peroxidation marker, in four different subject groups. [58]. Elevated levels of MDA in saliva were observed in healthy subjects with quid chewing/tobacco use, patients with potential malignant disorders (PMD) and OSCC groups compared to healthy subjects. These results suggest that salivary lipids likely play an important role in oral cancers and can be used as a diagnostic biomarker to detect PMD and OSCC.

Higher levels of glutathione peroxidase (GSHPx), an antioxidant enzyme, was also observed in patients with periodontitis. Therefore, increased levels of GSHPx and MDA, can indicate increased lipid peroxidation in patients with periodontal disease which is further elevated by smoking [41]. Using a targeted lipidomic approach, increased levels of salivary isoprostanes were evaluated in association with periodontal disease status with and without additional cigarette smoking. The levels of salivary prostaglandins E2 (PGE2), D2 (PGD2), and F2*α* (PG F2*α*) have been successfully used as biomarkers for chronic inflammatory processes and to assess the degree of oxidative stress caused by smoking and periodontitis. The results have shown that there was a significant redox alteration and fatty acid metabolism caused by periodontitis [59].

Changes in cellular lipid metabolism which occur in the early stages of tumour progression are commonly seen in most solid tumours. However, it is not clear if the tumour-specific altered synthesis and metabolism of lipids can be detected at the level of systemic lipid metabolism. For example, malignant cells may have a different lipid metabolism from healthy cells. Increased lipogenesis is commonly seen in most solid tumours and may also affect the cells' sensitivity to chemotherapy. Hilvo et al. [60] have associated the altered serum level of monounsaturated oleic acid with response to chemotherapy in breast cancer patients. Triacylglycerols containing mostly oleic acid (C18 : 1) were found in decreased levels in patients with pathologic complete response. Some of these triacylglycerols were related with estrogen receptor status and with disease survival outcomes of the patients. These results suggest that the changes in the serum levels of oleic acid in breast cancer patients can be related to their response to chemotherapy. Using a HeLa cell line, Li and Yuan [61] suggested that phospholipids and related phospholipases play important roles in paclitaxel-induced apoptosis in HeLa cells. Recently, Laus et al. [62] found that patients with advanced head and neck squamous cell carcinoma that responded to an organ preservation protocol had a different saliva lipid profile from those patients that did not respond. These studies clearly suggest that lipidomics research can determine and provide new understandings for the molecular mechanisms of the disease and its comprehensive analysis can lead to new biological marker discovery.

### 3.4. Salivary Lipid Profile in Correlation to Serum Lipid Profile

Karjalainen et al. measured the cholesterol concentration in the saliva of healthy adults and concluded that salivary concentration levels reflect cholesterol levels in serum [48]. Cholesterol and its esters, mono-, di-, and triglycerides, free fatty acids, together with a small amount of phospholipids represent the main lipid components in saliva. A study of serum and saliva lipids in about 100 healthy subjects showed that there is a reasonable correlation between the serum and saliva concentration of total cholesterol (TC), triglycerides (TGL), high density lipoprotein cholesterol (HDLC), and very low density lipoprotein cholesterol (VLDLC), indicating that saliva can be used as a noninvasive diagnostic fluid for lipid analysis [63].

### 3.5. Serum Lipid Level and Periodontal Status

The increased incidence of periodontal diseases in patients with atherosclerosis is related to acute and chronic coronary heart disease. Periodontitis seems to cause alteration in salivary total cholesterol (TC), high-density lipoprotein (HDL), low-density lipoprotein (LDL), and triglyceride (TG) levels. Thus, there is a causal association between chronic periodontitis and salivary lipid profiles. Another possible explanation for the mechanism that relates periodontitis to cardiovascular disease is that the release of bacteria and bacterial products from the chronic periodontal lesion into the blood stream leads to a systemic inflammatory response, which is a risk factor for a cardiovascular disease [64]. The study of salivary lipid profiles [65] has shown that there is an association between the chronic periodontitis and salivary lipid levels. The mean difference in the concentrations of TC and TG in saliva of patients with chronic periodontitis was statistically significant compared to the saliva of healthy patients. Concentrations of HDL and LDL were not statistically significant, but there was a difference in their mean levels. Concentration of LDL was higher in saliva of patients with chronic periodontitis, while the mean levels of HDL were high in the healthy patients. Increased salivary lipids in saliva of chronic periodontitis patients suggest an association between hyperlipidemia and periodontitis. Cardiovascular diseases and periodontitis have several risk factors in common. Since hyperlipidemia is a main risk factor for cardiovascular diseases, such as atherosclerosis, cardiac ischemic disorders, and strokes, it is imperative to determine its causes.

## 4. Analytical Methods

Analytical methods used in lipidome analysis differ significantly from metabolomic methods. The physicochemical diversity of the metabolome is enormous as demonstrated by partition coefficient values that range over 40 orders of magnitude [64]. Water soluble sugars and completely water insoluble lipids such as triglycerides are both an integral part of the metabolome. This diversity has led to different strategies for developing subomics analytical methods like lipidomics.

Lipidomic analysis involves lipid extraction from the sample and then lipid identification and quantification, with or without previous analytical separation, using chromatographic methods. The major challenge in lipidomic analysis is lipid extraction from the sample. Another challenge is lipid identification in lipid analysis due to the diverse nature of lipid species. Most lipid molecules are amphiphilic or amphipathic molecules, with a hydrophilic or polar ionic group, and nonpolar hydrophobic fatty acyl chain(s). Their specific physicochemical properties must be considered during sample preparation, chromatographic separation, and ionization in the MS detector. Due to their hydrophobic and amphipathic nature, and diversity in structural and physicochemical properties, a single extraction method may not yield the complete lipidome.

### 4.1. Common Extraction Methods Used in Lipidomics

Human saliva contains many electrolytes (bicarbonate, calcium, chloride, potassium, sodium, magnesium, and phosphate) and a variety of proteins represented by enzymes (i.e., *α*-amylase, maltase, and peptidases), immunoglobulins, and other antimicrobial factors, mucosal glycoproteins, traces of albumin, and some polypeptides and oligopeptides [66], which might interfere with the analysis. Established protocols for extraction of lipids involve two-phase liquid-liquid extractions after one-phase protein precipitation and removal by centrifugation. Protein removal by simultaneous protein precipitation with organic solvents used for lipid extraction is the easiest and the most convenient method. The subsequent introduction of a two-phase system by the addition of water and/or organic solvent is used to separate hydrophilic and hydrophobic metabolites. Additional washing steps that are used to increase lipid recovery makes the process time-consuming and hard to automate.

The aim of lipid extraction is to separate as many lipids as possible from their nonlipid components. Nonpolar organic solvents in which lipids are soluble like chloroform, methyl-*tert*-butyl ether, and heptane, are typically used to extract lipids from the aqueous phase. For example, extraction using a chloroform/methanol/H_2_O solvent mixture has been found to be effective and is a widely used method for extracting lipids from aqueous analytes [67]. The upper polar phase mainly consists of methanol and water, whereas the bottom (nonpolar) phase is rich in chloroform, and lipids dissolve there. Proteins usually accumulate at the interface between the two. After phase separation, lipids are found in the chloroform phase. However, chloroform is quite toxic and collection of the lower chloroform layer, after phase separation, cannot be easily automated. The chloroform based-extraction method has been recently replaced with modified methods using less hazardous methyl-*tert*-butyl ether (MTBE) [68] and butanol–methanol (BUME) [69] nonpolar organic solvents. Extraction with MTBE/methanol/water eliminates some of the problems of extraction with chloroform since MTBE is in the top layer, enabling it to be more easily removed, making the extraction process to be easily automated. However, the MTBE layer contains a significant amount of aqueous components that may carry over water-soluble contaminants, while the BUME method has less water-soluble contaminants present in the organic layer. Theodoridis et al. recommended the removal of particulate matter before protein precipitation using solvents such as acetonitrile for LC-MS-based metabolomics [70]. The addition of acetonitrile in the ratio of 2 : 1 to a biofluid sample was found to be most efficient in removing protein from the sample (efficiency > 96%) [71]. However, none of the extraction methods leads to complete extractions of all lipid species. Acidic lipids, for example, can be efficiently extracted only in acidic conditions, but the use of acidic or alkaline conditions may induce hydrolysis of certain lipids.

### 4.2. Lipidomic Analysis

The high structural diversity and an enormous number of different lipid compounds present in the lipidome, together with wide concentration ranges, pose a significant analytical challenge (i.e., effective separation and highly sensitive detection methods) for lipidomic analysis. Many of lipid metabolites are closely related isomers (e.g., position of the acyl chain and position and geometry of the double bond), and even though they exhibit strong chemical and structural similarity, their biological activity can be very different. Being able to detect variation in linkages and positions of fatty acyl chain(s) on the lipid backbone, functional group modification, and occurrence of the molecular species as isomers or isobars are among some of the greatest analytical challenges in lipidomic analysis. Traditional methods of lipid analysis such as thin layer chromatography (TLC), gas chromatography (GC), and nuclear magnetic resonance spectroscopy (NMR) are limited by lower sensitivity and accuracy.

The commonly used methods for lipidomic analysis are gas chromatography, after derivatization of polar groups, or liquid chromatography hyphenated to mass spectrometry (GC-MS or LC-MS). GC-MS is a well-established technique for the analysis of fatty acid methyl esters after the esterification of all lipids. Since GC requires the compounds to be thermally stable and to have high vapour pressure to volatilize during injection, sample derivatization is necessary. Fatty acid chains are first cleaved by hydrolysis and then esterified to produce fatty-acid methyl esters (FAMEs). This approach provides information on fatty acid composition, but the information on intact lipids (individual lipid classes) is completely lost. Although GC exhibits good separation, it requires extensive sample preparation and time-consuming analysis.

Lipidomic methods have greatly advanced in recent years with a constant advance in mass spectrometry and with the development of highly sensitive MS detectors that provide high resolution, sensitivity, selectivity, and throughput, and can be coupled with chromatographic separation. Lipidomics analysis based on MS has been successful in identifying some lipid markers for early detection of disease. In MS, neutral analyte molecules are first ionized, with resulting charged species separated by magnetic and/or electric fields, according to their mass-to-charge ratio (*m*/*z*). A mass spectrum is constructed with the ion signal intensity plotted against *m*/*z*.

However, MS is not without problems due to interference, especially through ion suppression effects. Ion suppression is one form of matrix effect that negatively affects accuracy, precision, and detection capability [72]. Consideration of ion suppression should be a part of the optimization and validation of quantitative lipidomics LC-MS methods. The ion-suppressing (or enhancing) agents caused by sample matrix, solvent or LC-MS system components, should be identified and quantified, with ion suppression effects either eliminated or reduced as much as possible. Differentiating between isomeric molecules often requires additional structural information or chromatographic separation prior to MS analysis.

Recent developments in lipid chromatography and high-accuracy high-resolution mass spectrometry made high throughput and quantitative analysis of the lipids feasible for the first time. Different strategies have been developed for comprehensive analysis of lipids in biological samples. MS analysis of lipids can be either chromatography based with LC separation prior to mass spectrometric analysis (LC-lipidomics) [73] or direct analysis without previous separation with mass spectrometry imaging (MSI) or in lipid extracts using shotgun lipidomics [74]. Thus, there are three main approaches used in lipidomic research:

- Liquid-phase separations coupled to MS (typically LC-MS)
- Direct infusion MS analysis (known as shotgun lipidomics)
- Direct analysis with desorption ionization MS approaches (mass spectrometry imaging (MSI))

The LC-MS approaches (reversed phase (RP), normal phase (NP), or hydrophilic interaction liquid chromatography (HILIC)) provide the platform for the most popular lipid analysis method. LC-MS provides retention time as an additional parameter that can help in the identification of lipid species. Technological advances in mass spectrometry and method development have produced infusion-based lipidomics or shotgun lipidomic analysis capable of characterizing lipid species by direct analysis of total lipid extracts. Shotgun lipidomics refers to the direct infusion of lipid species into the MS for targeted or global mass spectrometry detection yielding a single mass spectrum representative of the whole composition of lipids in the sample without prior chromatographic separation. Mass spectrometry imaging (MSI) techniques can also provide information on a spatial distribution of various biomarkers thus adding an extra dimension to that of conventional MS. Shotgun lipidomics, or direct infusion of the lipid extract, relies solely on MS techniques for lipid fingerprinting.

### 4.3. Untargeted and Targeted Lipidomics

MS-based lipidomics can be untargeted or targeted (e.g., “global” versus “targeted” analysis). Untargeted lipidomics are unbiased and used to detect as many lipids as possible without any prior information, while targeted lipidomics focuses on specific lipid classes and their fragmentation patterns. Untargeted lipidomics generate large amounts of data that are translated into lipid fingerprints that provide information on the simultaneous presence of many lipids and lipid metabolites. They are subsequently processed as a comparison between control and test sample material in order to detect observable differences between groups. However, quantitation is limited, and identification of unknown compounds is often the main challenge in untargeted metabolomics approach. Modern MS can detect thousands of lipids and lipid metabolites with high accuracy and precision. Fourier-transform ion cyclotron resonance MS (FT-ICR MS) and time-of-flight MS (ToF-MS) can provide extremely high mass accuracy of 0.1-1.0 ppm [75] and within 10 ppm [76], respectively. This high accuracy in mass for any detected fragment and abundant isotopic information in the fragmentation spectra enables us to assign unique elemental composition of the starting compound and provide information about its structure in complex mixtures.

Modern ionization methods, such as atmospheric pressure chemical ionization (APCI), electrospray ionization (ESI), matrix-assisted laser desorption ionization (MALDI), and other derivative methods, have improved the ionization and fragmentation of lipid molecules to resolve complex mixtures of lipids and identify their unique constituents. High-resolution tandem mass spectrometry (MS/MS or MS^2^) allows direct analysis of isomeric steroid metabolites. Tandem mass spectrometry (MS/MS) is a well-proven technique for the identification and quantitation of lipids because there are many isobaric lipid species which are not separated by direct infusion MS. In tandem mass spectrometry, compounds are separated by their mass-to-charge (*m*/*z*) ratio by the first mass spectrometer (MS1), fragmented as they exit, and identified based on their fragments by a second mass spectrometer (MS^2^). Precursor ions of a specific *m*/*z* ratio are selected (MS1) and then fragmented (MS2) to generate a product ion for detection. Therefore, tandem MS involves three distinct steps of selection-fragmentation-detection. The product ions (fragments) are used to describe features of the chemical structure of the precursor ion.

In shotgun lipidomics, an organic solvent extract is analyzed directly by soft ionization techniques such as electrospray ionization-mass spectrometry (ESI-MS) and MALDI-MS that can transform biomolecules into ions, or with tandem MS/MS in multiple scan modes (product ion, neutral loss, precursor-ion, and selective/multiple reaction monitoring) [77]. MALDI along with ESI allows for ionization and measurement of large molecular weights that could not be done before with classic ionization methods. The analysis can be designed as relative or absolute quantitative assays by using chemometric methods to process mass spectrometer intensity data. Shotgun lipidomics by hybrid quadrupole time-of-flight mass spectrometry has allowed absolute quantification of hundreds of intact molecular species including glycerophospholipid species, glycerolipid species, sphingolipid species, and sterol lipids [74]. A few researchers claim that chromatographic separation is no longer required and that it can be replaced with multidimensional MS-based shotgun lipidomics (MDMS-SL). Chromatographic separation is, in this case, replaced with intrasource separation and by exploiting unique chemical structures of different lipid classes. There are 4 components in MDMS-SL analysis: (1) multiplexed extractions under different extraction conditions for sample preparation, (2) intrasource separation of lipids based on their electrical properties to resolve lipid classes and mass spectrometry, (3) identification of individual molecular species of the selectively ionized lipids utilizing multidimensional MS identification, and (4) two-step quantification/processing of the identified individual molecular species with internal standards. However, mass spectra complexity, ion suppression in the source, and common presence of lipid isomers justify the use of chromatographic separation with MS [78].

## 5. Limitations in Lipidome Analysis

Tandem mass spectrometry (MS/MS) has led to great advancements in lipid analysis, affording characterization of complex lipid structures. However, complete structural characterization of complex lipids, such as glycerophospholipids, by tandem mass spectrometry (MS/MS) is still a major challenge. MS is limited in its ability to distinguish between many structural isomers commonly found among lipids, like isomeric eicosanoid species, positional isomers of inositol phosphates in polyphosphoinositides, phospholipids with modified fatty acids, a variety of sphingoids, and numerous intermediate metabolites. Lipid identification via MS analysis cannot determine (a) the stereochemistry, (b) double bonds' location and geometry, and (c) the site of fatty acid attachment on glycerol in glycerolipids and glycerophospholipids. Positions of double bonds in unsaturated fatty acids can have markedly different effects on biological function and serve as biomarkers of disease pathology. Furthermore, coverage of the entire cellular lipidome is still an ambition. Moreover, an understanding of the biochemical mechanisms responsible for a disease state is rarely definite.

## 6. Conclusions

There is much interest in the comprehensive analysis of salivary lipids to identify lipid biomarkers that can be used for disease diagnosis and for monitoring disease progression. Salivary lipids are among the most essential cellular components in human saliva and play a main role in many biological processes (e.g., energy storage, cellular structure, and cell signalling). Although salivary lipid composition changes in various pathological states and diseases, little is known about the role and composition of salivary lipids and about their interaction with other important ingredients of human saliva, including proteins, glycoproteins, and salivary mucins. Many specialized sensitive analytical methods involving MS have been developed to identify and monitor changes in these lipid biomarkers. These methods show great promise; however, much more work is required before it can become an effective diagnostic and disease management tool.

## Figures and Tables

**Table 1:** Classification of lipids.

Category	Abbreviation	Example
Fatty acyls	FA	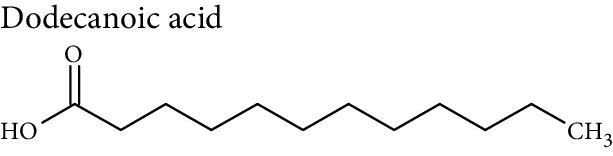
Glycerolipids	GL	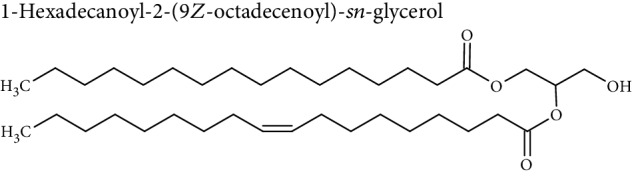
Glycerophospholipids	GP	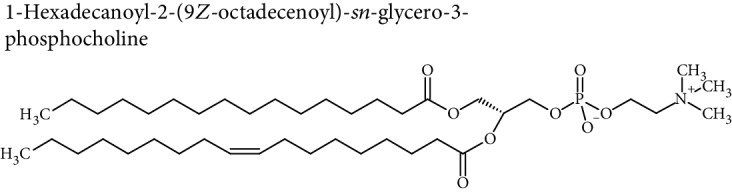
Sphingolipids	SP	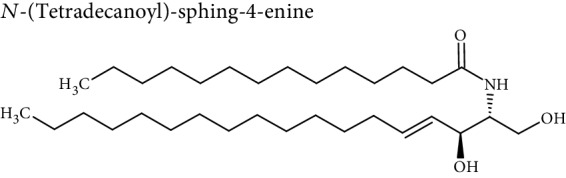
Sterol lipids	ST	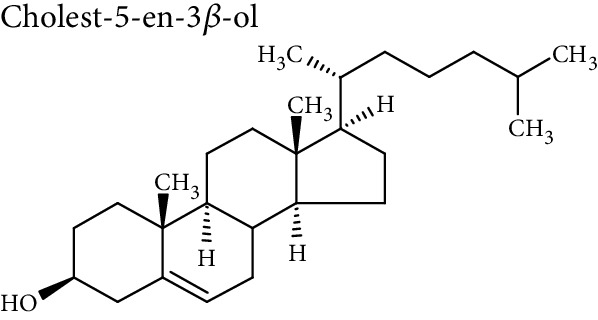
Prenol lipids	PR	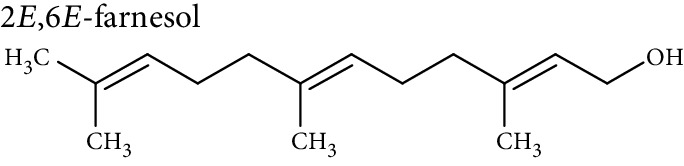
Saccharolipids	SL	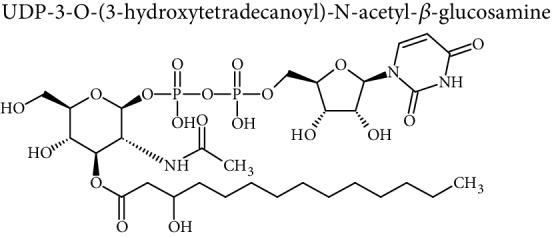
Polyketides	PK